# Mechanical Stress Activates Smad Pathway through PKCδ to Enhance Interleukin-11 Gene Transcription in Osteoblasts

**DOI:** 10.1371/journal.pone.0013090

**Published:** 2010-09-29

**Authors:** Shinsuke Kido, Rika Kuriwaka-Kido, Yuka Umino-Miyatani, Itsuro Endo, Daisuke Inoue, Hisaaki Taniguchi, Yasumichi Inoue, Takeshi Imamura, Toshio Matsumoto

**Affiliations:** 1 Department of Medicine and Bioregulatory Sciences, The University of Tokushima Graduate School of Medical Sciences, Tokushima, Japan; 2 The 21st Century Center of Excellence (COE) Program, The University of Tokushima Graduate School of Medical Sciences, Tokushima, Japan; 3 Department of Obstetrics and Gynecology, The University of Tokushima Graduate School of Medical Sciences, Tokushima, Japan; 4 Division of Molecular Enzyme Physiology, University of Tokushima Institute for Enzyme Research, Tokushima, Japan; 5 Division of Biochemistry, The Cancer Institute of the Japanese Foundation for Cancer Research (JFCR), Tokyo, Japan; The University of Akron, United States of America

## Abstract

**Background:**

Mechanical stress rapidly induces ΔFosB expression in osteoblasts, which binds to *interleukin (IL)-11* gene promoter to enhance IL-11 expression, and IL-11 enhances osteoblast differentiation. Because bone morphogenetic proteins (BMPs) also stimulate IL-11 expression in osteoblasts, there is a possibility that BMP-Smad signaling is involved in the enhancement of osteoblast differentiation by mechanical stress. The present study was undertaken to clarify whether mechanical stress affects BMP-Smad signaling, and if so, to elucidate the role of Smad signaling in mechanical stress-induced enhancement of IL-11 gene transcription.

**Methodology/Principal Findings:**

Mechanical loading by fluid shear stress (FSS) induced phosphorylation of BMP-specific receptor-regulated Smads (BR-Smads), Smad1/5, in murine primary osteoblasts (mPOBs). FSS rapidly phosphorylated Y311 of protein kinase C (PKC)**δ**, and phosphorylated PKCδ interacted with BR-Smads to phosphorylate BR-Smads. Transfection of PKCδ siRNA or Y311F mutant PKCδ abrogated BR-Smads phosphorylation and suppressed *IL-11* gene transcription enhanced by FSS. Activated BR-Smads bound to the Smad-binding element (SBE) of *IL-11* gene promoter and formed complex with ΔFosB/JunD heterodimer via binding to the C-terminal region of JunD. Site-directed mutagenesis in the SBE and the AP-1 site revealed that both SBE and AP-1 sites were required for full activation of *IL-11* gene promoter by FSS.

**Conclusions/Significance:**

These results demonstrate that PKCδ-BR-Smads pathway plays an important role in the intracellular signaling in response to mechanical stress, and that a cross-talk between PKCδ-BR-Smads and ΔFosB/JunD pathways synergistically stimulates IL-11 gene transcription in response to mechanical stress.

## Introduction

Mechanical stress to bone plays a crucial role in maintaining bone homeostasis. Immobilization, long-term bed rest, or microgravity in space causes a marked loss of bone mass and strength, due to reduced bone formation as well as enhanced bone resorption [1]–[4]. Although the enhanced bone resorption can be inhibited by a treatment with bisphosphonates [4], [5], it has been difficult to stimulate the unloading-induced suppression of bone formation. Therefore, it is important to clarify the mechanism whereby bone formation is suppressed by mechanical unloading.

Mechanical stress to bone causes a rapid fluid flow surrounding osteoblasts and osteocytes, and elicits fluid shear stress (FSS) to these cells. FSS is shown to be one of the most important signal transduction mechanisms to enhance osteoblastic differentiation and bone formation in response to mechanical loading to bone [6], [7]. FSS rapidly stimulates an intracellular signaling cascade in cells of the osteoblast lineage: stimulation of gadolinium-sensitive Ca channel with an increase in intracellular calcium, activation of extracellular signal-regulated kinase (ERK), and phosphorylation of cyclic AMP response element-binding protein (CREB) by ERK [8]–[13]. We have previously demonstrated that phosphorylated CREB binds to *fosB* gene promoter, causing an enhancement of *fosB* gene transcription and an increase in ΔFosB expression [14], and that ΔFosB forms a heterodimer with JunD on *Interleukin (IL)-11* gene promoter to enhance IL-11 expression [13]. The expression of IL-11 in osteoblastic cells is reduced by mechanical unloading [13] and aging [15], and is enhanced by mechanical loading [13]. Furthermore, transgenic mice overexpressing IL-11 show high bone mass with continued increase of bone mineral density with aging due to an enhanced bone formation without an increase in bone resorption [16]. These observations suggested to us that IL-11 mediates mechanical stress signals to osteoblast differentiation signal.

Bone morphogenetic proteins (BMPs) play pivotal roles in the regulation of osteoblast differentiation and bone formation [17], [18]. When artificially implanted into muscle tissues, BMPs induce ectopic bone formation. However, the role of BMPs in mediating mechanical stress signal to osteoblastogenic signal remains unclear. BMP signals are transmitted via phosphorylation by type I BMP receptor of BMP-specific receptor-regulated Smads (BR-Smads) including Smad1, 5 and 8. Phosphorylated BR-Smads then form heteromeric complex with Smad4, a common Smad, and translocate into the nucleus, where they regulate transcription of various target genes [19]-[22]. Because our preliminary experiments demonstrated that not only mechanical stress but also BMP-2 stimulate IL-11 expression in osteoblastic cells (Figure S1), there is a possibility that BR-Smad signaling is also involved in the enhancement of osteoblast differentiation in response to mechanical stress. In order to address this issue, we investigated the effect of mechanical stress on BR-Smad phosphorylation as well as the interaction of BR-Smads with activator protein (AP)-1 transcription factors and *IL-11* gene promoter in osteoblastic cells. The results demonstrate that mechanical stress by FSS to murine primary osteoblasts (mPOBs) *in vitro* stimulates Smad1/5 phosphorylation, that mechanical stress-induced phosphorylation of Smad1/5 is mediated via phosphorylation and activation of protein kinase C (PKC)**δ**, that activated Smad1/5 forms complex with ΔFosB and JunD on *IL-11* gene promoter, and that *IL-11* gene transcription is cooperatively stimulated by ΔFosB/JunD and Smad1/5 in response to mechanical stress.

## Results

### Mechanical stress induces phosphorylation of BR-Smads in osteoblasts

**Figure 1:**
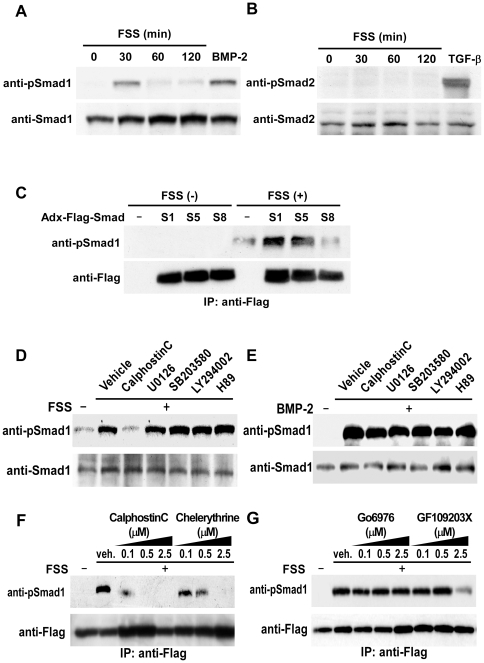
Mechanical stress induces phosphorylation of BR-Smads via PKC. (A and B) Experiments were performed by culturing mPOBs for 48 h and then exposing them to FSS for the indicated times. Phosphorylation of endogenous BR-Smads using an antibody against phosphorylated Smad1 that cross-reacts with phosphorylated Smad5/8 (A), and endogenous Smad2 using an antibody against phosphorylated Smad2 that cross-reacts with phosphorylated Smad3 (B) was analyzed by Western blot analysis. Proteins from 4 wells were analyzed in each time point, and the experiments were repeated for 3 times with similar results. Results from a representative experiment were presented. (C) Flag-Smad1, Flag-Smad5 or Flag-Smad8 in adenovirus vector was infected into mPOBs, and mPOBs were cultured as above. They were then exposed to FSS for 30 min and phosphorylation of exogenous Smad1/5/8 was analyzed by Western blot analysis using an antibody against phosphorylated Smad1 that cross-reacts with phosphorylated Smad5/8. Proteins from 4 wells were analyzed in each lane, and the experiments were repeated for 3 times with similar results. Results from a representative experiment were presented. (D and E) mPOBs were pretreated with vehicle, 10 µM calphostin C, 10 µM U0126, 10 µM SB203580, 10 µM LY294002, or 10 µM H89 for 30 min and then exposed to FSS for 30 min (D). mPOBs were treated in the same way in the presence of 300 ng/ml BMP-2 (E). Phosphorylation of endogenous Smad1/5 was analyzed by Western blot analysis. Proteins from 4 wells were analyzed in each lane, and the experiments were repeated for 3 times with similar results. Results from a representative experiment were presented. (F and G) In order to examine dose-dependent suppression of BR-Smad phosphorylation by various PKC inhibitors, mPOBs were infected with an adenovirus expressing Flag-Smad1 for 48 hours. mPOBs were pretreated with the indicated doses of calphostin C or chelerythrine (F), or the indicated doses of Go6976 or GF109203X (G) for 30 min, and then exposed to FSS for 30 min. Cells were homogenized and Flag-Smad1 was immunoprecipitated using an anti-Flag antibody. Phosphorylation of exogenous Smad1 was analyzed by Western blot analysis. Proteins from 4 wells were analyzed in each lane, and the experiments were repeated for 2 times with similar results. Results from a representative experiment were presented.

BMP-Smad pathway plays a central role in osteoblast differentiation and bone formation. In order to clarify the contribution of BMP-Smad signaling in osteoblasts in response to mechanical stress, we first investigated the effect of FSS on phosphorylation of Smads. FSS *in vitro* to primary osteoblasts derived from newborn mice calvariae enhanced phosphorylation of BR-Smads, Smad1/5/8, within 30 minutes using an antibody against phosphorylated Smad1 that cross-reacts with phosphorylated Smad5/8 (Figure 1A). Because mechanical loading to osteoblasts does not alter the expression levels of BMP-2 (Figure S2), these results suggest that mechanical stress to osteoblasts enhances BR-Smads phosphorylation without stimulation by BMP. The effect of FSS on phosphorylation of transforming growth factor-β-specific R-Smads, Smad2/3, was also examined using an antibody against phosphorylated Smad2 that cross-reacts with phosphorylated Smad3. As shown in Figure 1B, FSS failed to induce phosphorylation of Smad2/3 in osteoblasts, showing that mechanical stress specifically phosphorylates BR-Smads in osteoblasts.

In order to clarify which BR-Smads were phosphorylated by FSS, we tested the effect of FSS on phosphorylation of exogenous Flag-Smad1, Flag-Smad5 or Flag-Smad8 in adenovirus vector infected into mPOBs. FSS induced phosphorylation of Smad1 and Smad5, but not of Smad8 in osteoblasts (Figure 1C). Because the antibody detects phosphorylated Ser463/465 of all BR-Smads and cannot differentiate among them, experiments using this antibody were interpreted as detecting phosphorylation of endogenous Smad1/5 in response to FSS.

### Induction of BR-Smad phosphorylation by mechanical stress is dependent on PKC

In order to clarify intracellular signaling pathways that lead to phosphorylation of Smad1/5 by FSS, the effect of inhibitors of PKC, mitogen-activated protein kinase, phosphatidylinositol-3-OH kinase (PI3K), and protein kinase A (PKA) was examined using concentrations that can inhibit the activity of the respective enzymes. mPOBs were exposed to a PKC inhibitor calphostin C, a mitogen-activated protein kinase kinase inhibitor U0126, a p38 kinase inhibitor SB203580, a PI3K inhibitor LY294002, or a PKA inhibitor H89, and then subjected to FSS. Only a pan-specific PKC inhibitor, calphostin C, abolished phosphorylation of Smad1/5, whereas any of the other kinase inhibitors, U0126, SB203580, LY294002 or H89, failed to inhibit Smad1/5 phosphorylation by FSS (Figure 1D). Neither calphostin C nor other inhibitors affected BMP-induced phosphorylation of Smad1/5 (Figure 1E). These results are consistent with the notion that the mechanism of FSS-induced BR-Smad phosphorylation is different from that of BMP, and that PKC is involved in this process.

We next examined the effect of conventional PKC (cPKC) inhibitors with different dose-dependent inhibitory effects on other classes of PKC, chelerythrine, Go6976, and GF109203X, on FSS-induced phosphorylation of Smad1. High doses of chelerythrine and GF109203X inhibited Smad1 phosphorylation stimulated by FSS (Figure 1F and 1G), while Go6976 did not affect FSS-induced phosphorylation of Smad1 even at the highest dose (Figure 1G). Because chelerythrine and GF109203X at high doses are reported to act as pan-specific inhibitors for PKC [23], [24], these observations suggest that PKC other than cPKC induces BR-Smad phosphorylation in response to FSS.

### PKCδ is activated by mechanical stress and phosphorylates BR-Smads

**Figure 2:**
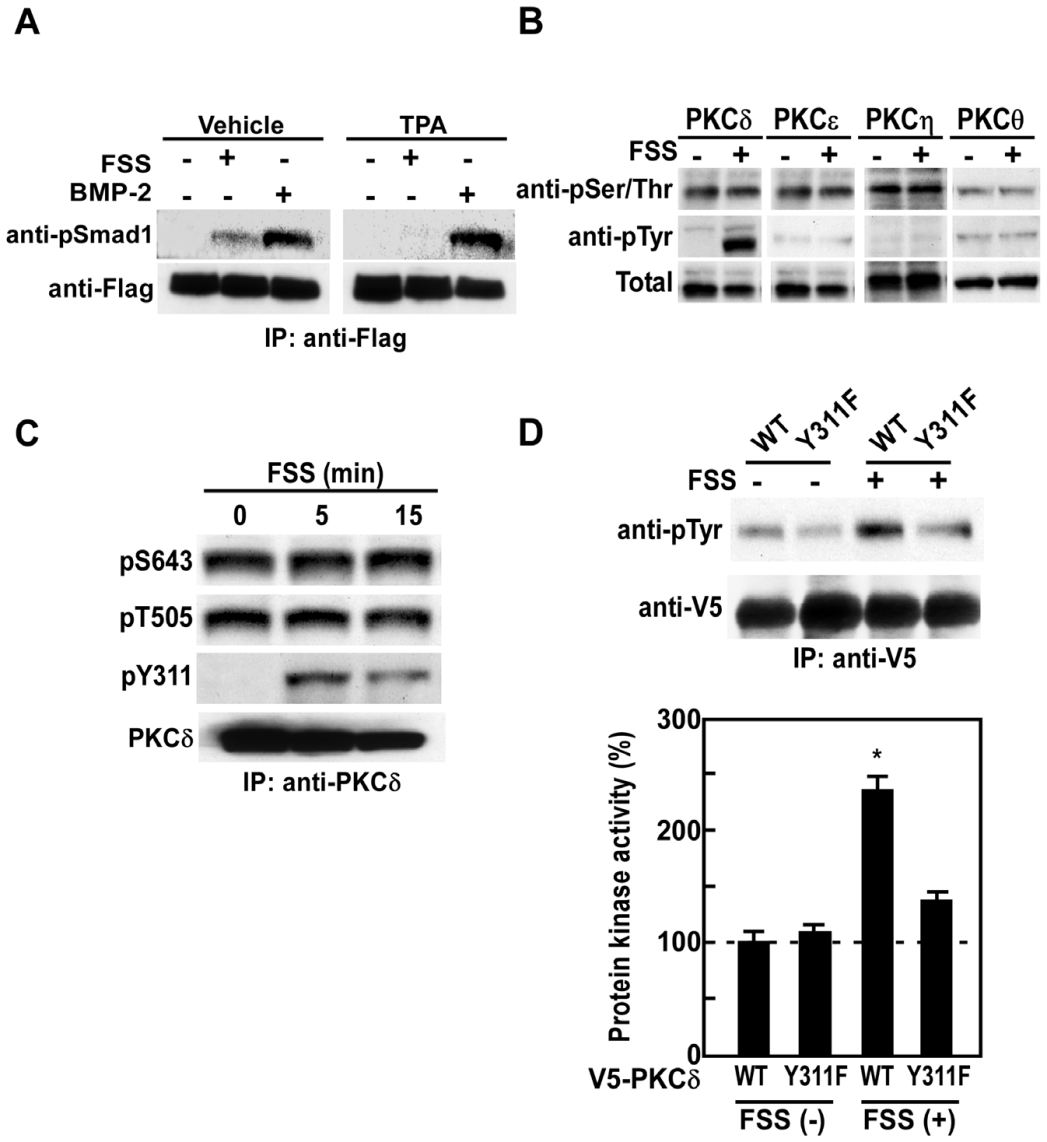
Mechanical stress activates PKCδ by Y311 tyrosine phosphorylation. (A) mPOBs were infected with an adenovirus expressing Flag-Smad1 for 48 hours, and were pretreated with vehicle or 100 nM TPA for overnight, and then exposed to FSS for 30min or treated with 300 ng/ml BMP-2. Phosphorylation of exogenous Smad1 was analyzed by Western blot analysis. Proteins from 4 wells were analyzed in each lane, and the experiments were repeated for 3 times with similar results. Results from a representative experiment were presented. (B) mPOBs were exposed to FSS for 30 min, and then PKCδ, PKCε, PKCη and PKCθ were immunoprecipitated from 500 µg of total cell lysates obtained from 4 dishes using antibodies against PKCδ, PKCε, PKCη and PKCθ.∼ Western blot analysis was performed using anti-phosphorylated serine/threonine and tyrosine antibodies. The experiments were repeated for 3 times with similar results. (C) mPOBs were exposed to FSS for the indicated times, and then phosphorylation at S643, T505, and Y311 of PKCδ was analyzed by Western blot analysis. (D) Mutant PKCδ with modification of Y311 to F311 (Y311F) was transiently transfected into mPOBs, and then cells were exposed to FSS for 30 min. In the upper panel, tyrosine phosphorylation was analyzed by Western blot analysis. Proteins from 4 wells were analyzed in each lane, and the experiments were repeated for 3 times with similar results. Results from a representative experiment were presented. In the lower panel, protein kinase activity was measured by kinase assay. The data were means ± S.E.M. for three experiments, and difference among the groups was analyzed by one-way ANOVA. *p<0.05 compared with the wild type without FSS.

PKC family members are divided into three species; cPKC, novel PKC (nPKC) and atypical PKC (aPKC). Because cPKC and nPKC but not aPKC are activated by diacylglycerol, and because cPKC and nPKC can be down-regulated by a pretreatment with phorbol esters [25], the effect of a phorbol ester, 12-O-tetradecanoylphorbol 13-acetate (TPA), on Smad1 phosphorylation induced by BMP or FSS was examined. Pretreatment with TPA inhibited FSS-induced Smad1 phosphorylation, but did not inhibit BMP-induced Smad1 phosphorylation (Figure 2A), showing that mechanical stress induces phosphorylation of BR-Smads through an activation of nPKC.

We thus examined the effect of FSS on the activation of nPKCs, including PKCδ, PKCε, PKCη and PKCθ. When FSS was applied to mPOBs and serine/threonine or tyrosine phosphorylation of the respective nPKC was examined, only tyrosine phosphorylation of PKC**δ** was enhanced by FSS (Figure 2B). In contrast, none of the other nPKCs, PKCε, PKCη or PKCθ, showed FSS-dependent phosphorylation of serine/threonine or tyrosine. Tyrosine phosphorylation at amino acid 311 (Y311) is critical for the activation of PKCδ [26], and Western blot analysis using an antibody against phosphorylated Y311 of PKCδ revealed that Y311 of PKCδ was phosphorylated by FSS (Figure 2C). Furthermore, overexpression of mutant PKCδ with modification of Y311 to F311 (Y311F) abrogated tyrosine phosphorylation of PKCδ by FSS (Figure 2D). These results demonstrate that PKCδ is specifically tyrosine-phosphorylated at Y311 by mechanical stress.

### Activated PKCδ by mechanical stress phosphorylates BR-Smads

**Figure 3:**
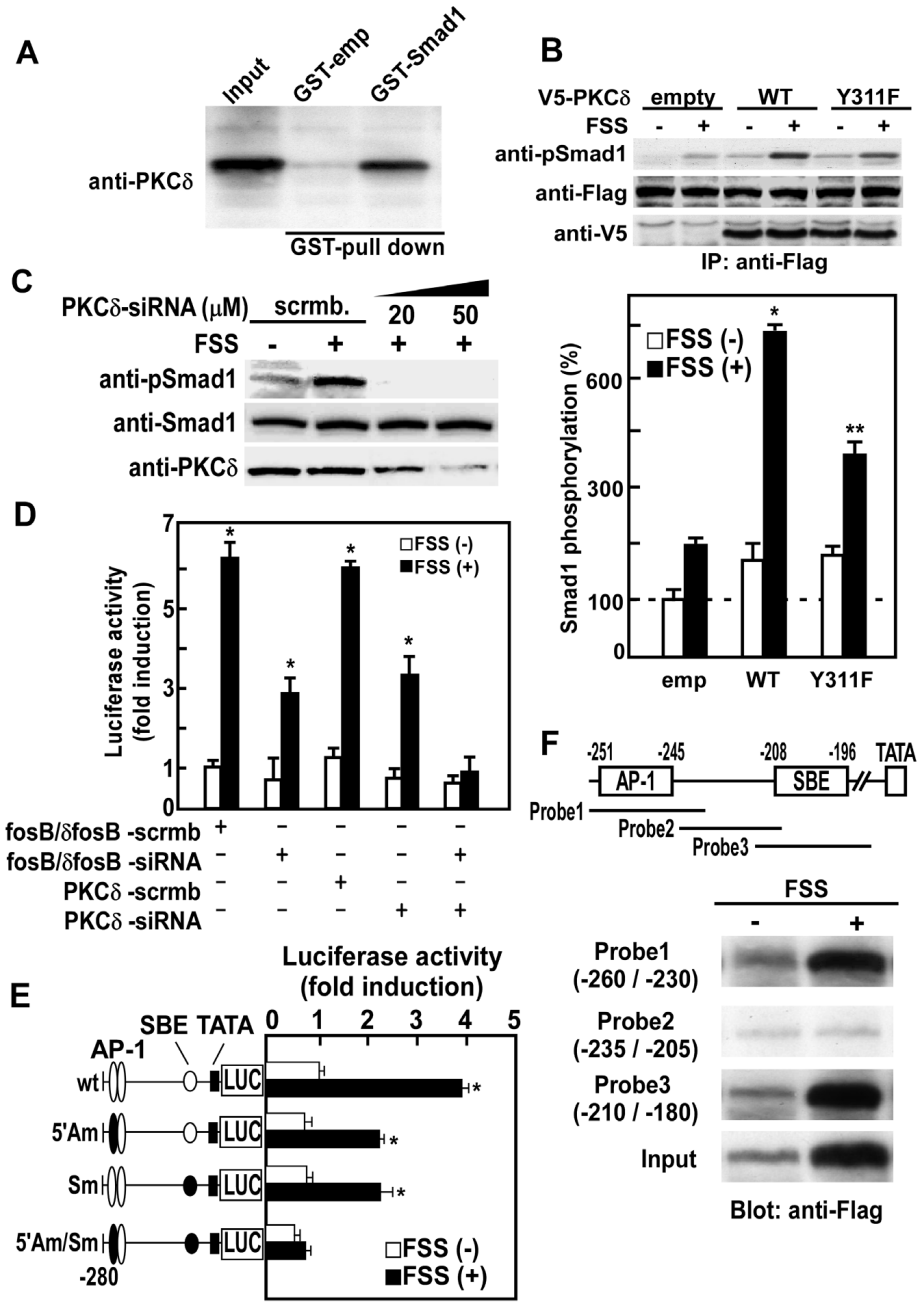
Mechanical stress-activated PKCδ phosphorylates BR-Smads, and phosphorylated BR-Smads interact with ΔFosB/JunD on *IL-11* gene promoter. (A) GST-Smad1 fusion protein was mixed with Glutathione-Sepharose and incubated at 4C° for 1 h. The Sepharose beads were then washed and used for GST pull-down assay. mPOBs were lysed in lysis buffer and aliquots of the lysate were then incubated under constant agitation for 1 h at 4C° with GST-Smad1 fusion protein coupled to Glutathione-Sepharose. Complexes were then washed three times and bound proteins were eluted and separated on SDS-PAGE. PKCδ bound to GST-Smad1 fusion protein was detected by Western blot analysis using an anti-PKCδ antibody. (B) Wild-type or Y311F mutant PKCδ were transiently transfected into mPOBs, and cells were exposed to FSS for 30 min. Phosphorylation of exogenous Smad1 was analyzed by Western blot analysis using an anti-phospho Smad1 antibody (upper panel). Proteins from 4 wells were analyzed in each lane, and the experiments were repeated for 3 times with similar results. Results from a representative experiment were presented. In the lower panel, the amount of exogenous phospho-Smad1 was quantitated and expressed as a percentage of the amount of phospho-Smad1 in mPOBs transfected with an empty vector without FSS. The data were means ± S.E.M. for three experiments, and difference between FSS(−) and FSS(+) in each group was analyzed by Student's *t* test. *p<0.01 and **p<0.05 compared with FSS(−). (C) Scrambled or indicated doses of PKCδ siRNA were transiently transfected into mPOBs, and then cells were exposed to FSS for 30 min. Phosphorylation of endogenous BR-Smads was analyzed by Western blot analysis using an antibody against phospho-Smad1 that cross-reacts with other BR-Smads. Proteins from 4 wells were analyzed in each lane, and the experiments were repeated for 3 times with similar results. Results from a representative experiment were presented. (D) Effect of FosB/ΔFosB and PKCδ knockdown on the induction of *IL-11* gene transcription by FSS was examined by transfecting mPOBs with siRNA oligonucleotide for FosB/ΔFosB and PKCδ and/or along with the luciferase reporter vector on *IL-11* gene promoter (−0.28K). Scrambled siRNA for each gene was used as an internal control. Cells were then exposed to FSS for 6 h and transcriptional activity was measured by dual-luciferase assay. Data were corrected for Renilla luciferase and expressed relative to FSS (−) cells. Values were expressed as the means ± S.E.M. for four experiments, and difference between FSS (−) and FSS (+) in each group was analyzed by Student's *t* test. *p<0.05 compared with FSS (−). (E) Deletion constructs of luciferase reporter including the 5′-flanking region of the wild-type mouse *IL-11* gene (WT), and substitution mutant constructs with mutations at the 5′AP-1 site (5′Am), SBE (Sm) or both 5′AP-1 and SBE (5′Am/Sm) were transiently transfected into mPOBs. Cells were then exposed to FSS for 6 h and transcriptional activity was measured by dual-luciferase assay. Data were corrected for Renilla luciferase and expressed relative to FSS (−) cells. Values were expressed as the mean ± S.E.M. for four experiments, and difference between FSS(−) and FSS(+) in each group was analyzed by Student's *t* test. *p<0.01 compared with FSS (−) cells. (F) Flag-tagged Smad1 was transfected into mPOBs. After mPOBs were subjected to FSS for 30 min, nuclear lysates were prepared and analyzed for DNA binding to various DNA probes (probe 1–3) of *IL-11* gene promoter by DNA precipitation assay. Flag-tagged Smad1 bound to DNA probes 1 to 3 was analyzed by Western blotting using an anti-Flag antibody. DNA probe 1 (−260 to −230) covers 5′AP-1 site (−251 to −245), DNA probe 3 (−210 to −180) covers SBE (−208 to −196), and DNA probe 2 (−235 to −205) does not cover either AP-1 site or SBE (upper panel). Not only DNA probe 3 (−210 to −180) covering SBE but also probe 1 (−260 to −230) covering upstream AP-1 site bound to Flag-tagged Smad1 in an FSS-dependent manner, whereas probe 2 did not bind to Smad1 (lower panel).

To investigate whether phosphorylated PKCδ by mechanical stress interacts with BR-Smads, we next performed Glutathione S-transferase (GST) pull-down assay. Western blot analysis using an anti-PKCδ antibody revealed that PKCδ binds to GST-Smad1 (Figure 3A), demonstrating that PKCδ physically interacts with Smad1.

In order to clarify whether Y311 phosphorylation of PKCδ can in fact activate PKCδ and phosphorylate BR-Smads, Smad1 phosphorylation by FSS was examined in mPOBs overexpressing wild-type or Y311F mutant PKCδ. Smad1 phosphorylation was robustly enhanced by FSS in osteoblasts overexpressing wild-type PKCδ, but was attenuated in osteoblasts overexpressing Y311F PKCδ (Figure 3B). Moreover, knockdown of PKCδ by small interfering RNA (siRNA) abrogated phosphorylation of endogenous Smad1/5 by FSS (Figure 3C). To further clarify whether Smad1/5 phosphorylation by PKCδ stimulates *IL-11* gene transcription in response to FSS, we examined the effect of PKCδ knockdown on *IL-11* gene promoter activity. Knockdown of PKCδ by siRNA markedly suppressed the enhancement of *IL-11* gene promoter activity by FSS, and FosB/ΔFosB knockdown to inhibit AP-1 signaling in combination with PKCδ knockdown almost completely abolished the stimulation of *IL-11* gene promoter activity by FSS (Figure 3D). Taken together, these observations demonstrate that mechanical stress induces phosphorylation of BR-Smads by activation via Y311 phosphorylation of PKCδ, which in turn enhances the expression of IL-11 in cooperation with AP-1 signal in osteoblasts.

### Phosphorylated BR-Smads by mechanical stress interact with ΔFosB/JunD on *IL-11* gene promoter

We have previously demonstrated that mechanical stress enhances IL-11 expression by an activation of *IL-11* gene promoter via heterodimer formation of ΔFosB and JunD [13]. In order to clarify whether there is any interaction between ΔFosB/JunD heterodimers and BR-Smads, we examined the effect of FSS on Smad1 binding to *IL-11* gene promoter as well as interaction of BR-Smads with ΔFosB/JunD heterodimers. Analysis of mouse *IL-11* gene promoter activity with site-directed mutagenesis at a putative Smad-binding element (SBE) (−208 to −196) and an upstream AP-1 binding site (−251 to −245) which confers *IL-11* gene transcription by ΔFosB/JunD [13] revealed that both SBE and AP-1 sites were required for full activation of *IL-11* gene promoter activity by FSS (Figure 3E). These observations can also explain why knockdown of PKCδ by siRNA only partially suppressed *IL-11* gene promoter activity (Figure 3D), while Smad1 phosphorylation by FSS was almost completely abrogated by siRNA (Figure 3C).

In order to examine whether BR-Smads bind to the SBE on *IL-11* gene promoter, Flag-tagged Smad1 was transfected to mPOBs and DNA precipitation assay was performed. Because mechanical stress to mPOBs enhanced Smad1 phosphorylation by PKCδ, transition of phosphorylated Smad1 into nucleus increased. As a result, the total amount of Smad1 in the nucleus shown as input in Figure 3F also increased by FSS. In addition to the increase in the total nuclear Smad1, not only DNA probe 3 (−210 to −180) covering SBE but also probe 1 (−260 to −230) covering upstream AP-1 site bound to Flag-tagged Smad1 in an FSS-dependent manner (Figure 3F). Probe 2 (−235 to −205) that does not cover either SBE or AP-1 site did not bind Flag-Smad1 (Figure 3F). These results demonstrate that FSS-activated Smad1 is incorporated into a complex with ΔFosB/JunD heterodimer on the *IL-11* gene promoter, and the complex is bound to both AP-1 site and SBE.

### BR-Smads form complex with ΔFosB/JunD heterodimer by binding to JunD

**Figure 4:**
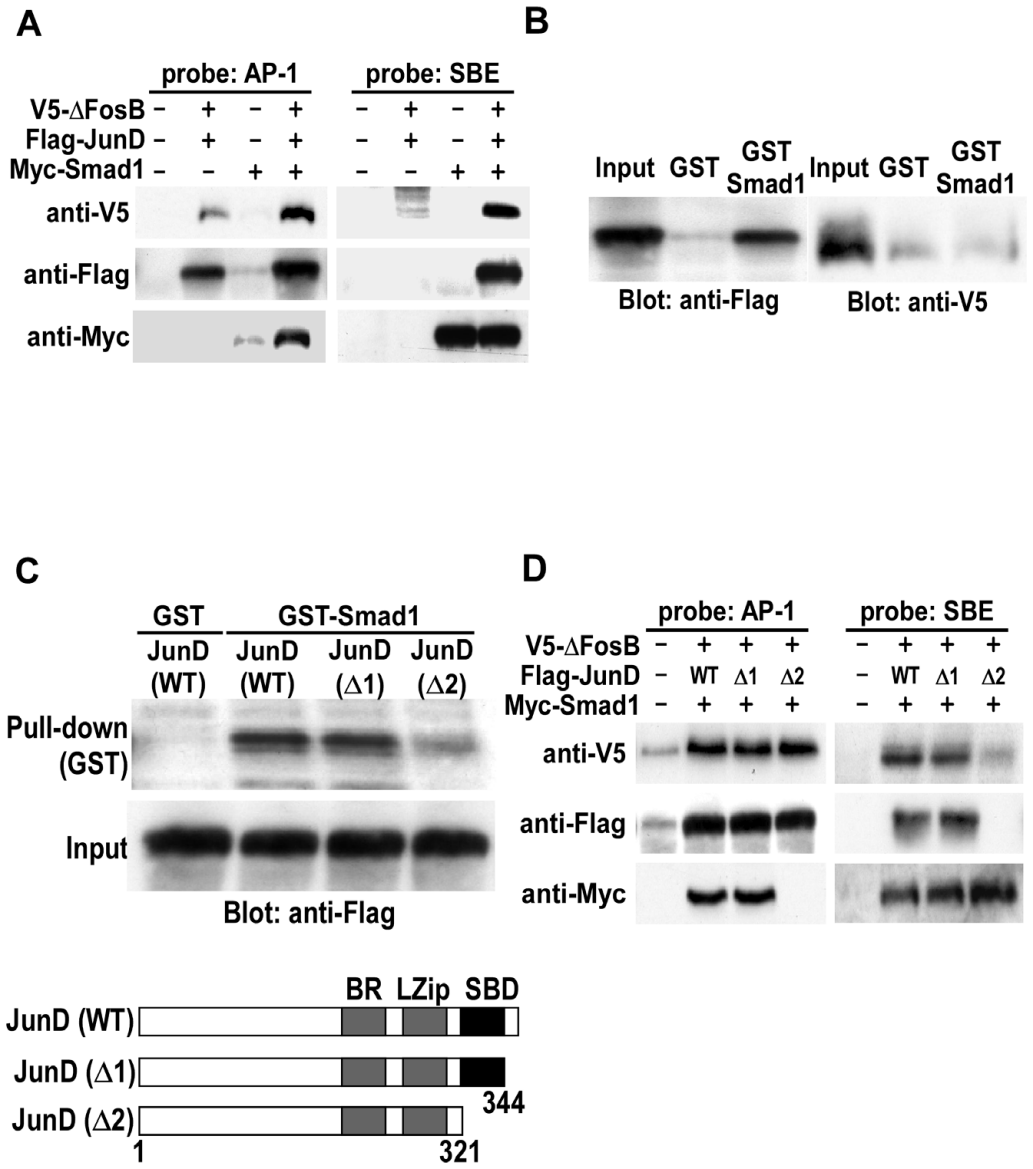
BR-Smads form complex with ΔFosB/JunD heterodimer by binding to JunD. (A) V5-tagged ΔFosB, Flag-tagged JunD and Myc-tagged Smad1 were co-transfected into mPOBs. Nuclear lysates were prepared and analyzed for binding of nuclear proteins to DNA probe 1 or 3 by DNA precipitation assay followed by Western blotting using antibodies against V5, Flag and Myc. Nuclear lysates from 4 wells were analyzed in each lane, and the experiments were repeated for 3 times with similar results. Results from a representative experiment were presented. (B) GST-Smad1 fusion protein was bound to Glutathione-Sepharose beads, and Flag-JunD or V5-ΔFosB was incubated with GST-Smad1 fusion protein coupled to Glutathione-Sepharose. Complexes were then washed and bound proteins were eluted and separated on SDS-PAGE. JunD or ΔFosB bound to GST-Smad1 fusion protein was detected by Western blot analysis using an anti-Flag or an anti-V5 antibody. (C) GST-Smad1 fusion protein was bound to Glutathione-Sepharose beads, and Flag-tagged WT or C-terminal truncated JunD was incubated with GST-Smad1 fusion protein coupled to Glutathione-Sepharose. Complexes were then washed and bound proteins were eluted and separated on SDS-PAGE. Flag-tagged WT or C-terminal truncated JunD bound to GST-Smad1 fusion protein was detected by Western blot analysis using an anti-Flag antibody (upper panel). Both JunD (WT) and JunD (1–344) (Δ1) had Smad-binding domain, but JunD (1–321) (Δ2) lacked C-terminal Smad-binding domain (lower panel). (D) V5-tagged ΔFosB and Flag-tagged JunD (WT), JunD (Δ1), or JunD (Δ2) along with Myc-tagged Smad1 were co-transfected into mPOBs. Nuclear lysates were prepared and analyzed for binding of nuclear proteins to DNA probe 1 or 3 by DNA precipitation assay, followed by Western blotting using antibodies against V5, Flag and Myc. Nuclear lysates from 4 wells were analyzed in each lane, and the experiments were repeated for 3 times with similar results. Results from a representative experiment were presented.

In order to clarify the relationship between Smad1 and ΔFosB/JunD heterodimer, V5-tagged ΔFosB, Flag-tagged JunD and Myc-tagged Smad1 were co-transfected into mPOBs and DNA precipitation assay was performed using upstream AP-1 probe (Probe 1) or SBE probe (Probe 3). V5-ΔFosB and Flag-JunD were bound to SBE when Myc-Smad1 was co-transfected, and Myc-Smad1 was bound to AP-1 when V5-ΔFosB and Flag-JunD were co-transfected (Figure 4A). These results demonstrate that Smad1 binds to SBE and ΔFosB/JunD heterodimer binds to AP-1 site on the *IL-11* gene promoter, and that Smad1 and ΔFosB/JunD heterodimer form complex on the *IL-11* gene promoter. In addition, a GST pull-down assay revealed that GST-Smad1 interacted with only Flag-JunD but not with V5-ΔFosB (Figure 4B), suggesting that Smad1 interacts with JunD to form a complex with ΔFosB/JunD heterodimer. To further delineate the interaction between Smad1 and ΔFosB/JunD heterodimer, we next searched binding domain of JunD to Smad1 by creating full length Flag-JunD, as well as deletion mutants of JunD, Flag-JunD(1–344) and Flag-JunD(1–321). Full length and JunD(1–344) with intact Smad-binding domain similarly bound to Smad1, but JunD(1–321) that lacks C-terminal Smad-binding domain failed to bind to Smad1 (Figure 4C). Consistent with these observations, full length JunD and JunD(1–344), but not JunD(1–321), interacted with SBE in the presence of Smad1 (Figure 4D). Thus, Smad1 forms complex with ΔFosB/JunD heterodimer by interacting with the C-terminal Smad-binding domain of JunD.

## Discussion

In this paper, we report a new signal crosstalk between PKCδ-Smads pathway and ERK-CREB-ΔFosB/JunD pathway in response to mechanical stress. Mechanical stress by FSS to osteoblasts enhances phosphorylation of BR-Smads via an activation of PKCδ. Phosphorylated BR-Smads form complex with ΔFosB/JunD heterodimers via binding to the C-terminal region of JunD, and BR-Smad binds to SBE and ΔFosB/JunD heterodimer binds to AP-1 site of the *IL-11* gene promoter. Consequently, they cooperate to lead to a full activation of the *IL-11* gene transcription.

In the present study, mechanical stress to osteoblasts transiently enhanced Smad1/5 phosphorylation without stimulation by BMP. Because BMP binding to its receptor phosphorylates BR-Smads by an activation of BMP receptor type I kinase, PKC inhibitors are not expected to inhibit BMP-induced phosphorylation of BR-Smads. However, in the present study, FSS-induced phosphorylation of BR-Smads was almost completely blocked by an inhibition of PKCδ, or transfection of Y311F mutant that cannot be phosphorylated by PKCδ at amino acid 311, or silencing PKCδ expression by PKCδ siRNA. These observations indicate that mechanical stress-induced stimulation of BR-Smad signaling is independent of BMP-BMP receptor signaling pathway, but is dependent upon PKCδ activation. Stimulation of PKC activity by mechanical stress to osteoblastic cells has been reported using cyclic stretch load by Flexercell system in calvarial osteoblasts [27]. However, it has been unknown which class of PKC is activated by mechanical stress to osteoblastic cells. The present study revealed that activation of PKCδ plays an important role for the phosphorylation and activation of BR-Smads by mechanical stress.

One of the earliest responses after mechanical loading to bone is an increase in the expression of c-Fos. However, because overexpression of c-Fos develops osteosarcoma without an enhancement of bone formation [28], c-Fos appears to enhance transformation instead of differentiation of osteoblasts. We previously demonstrated that mechanical stress to osteoblasts rapidly enhances the transcription of *fosB* gene with a similar time course to that of *c-fos* gene, and that the enhancement of *fosB* gene transcription is mediated via an activation of ERK-CREB signaling [14]. Activation of *fosB* gene transcription causes an increase in the expression of FosB and its truncated splice variant, ΔFosB. Because ΔFosB overexpression in mice causes an enhancement of bone formation [29], [30], the increase in ΔFosB in response to mechanical stress may play an important role in the stimulation of bone formation by mechanical stress. To this end, it is interesting to note that ΔFosB forms heterodimer with JunD on the AP-1 site of the *IL-11* gene promoter, and that the binding of ΔFosB/JunD heterodimer on the *IL-11* gene promoter enhances the promoter activity to increase the expression of IL-11 after mechanical stress [13]. However, AP-1 factors regulate the transcription of wide range of genes, and this signaling cascade in itself does not enhance a group of genes that are specific for osteoblast differentiation.

BMP regulates differentiation of various types of cells, but its main target is the regulation of osteoblast differentiation. Because BR-Smads mediate canonical BMP signaling, activation of BR-Smad signaling by mechanical stress via an activation of PKCδ may play an important role for the enhancement of osteoblastic differentiation by mechanical stress. In fact, inhibition of phosphorylation of BR-Smads by transfection of Y311F mutant of PKCδ or knockdown of PKCδ abrogated the stimulation of *IL-11* gene promoter activity by mechanical stress. These results are consistent with the notion that BR-Smads and ΔFosB/JunD heterodimer act synergistically to enhance osteoblast differentiation, and that both the activation of BR-Smads and the formation of ΔFosB/JunD heterodimer are required for a full activation of *IL-11* gene transcription in response to mechanical stress. Because IL-11 transgenic mice exhibit increased bone mass with enhanced bone formation without an increase in bone resorption [16], and because an increase in IL-11 expression causes a down-regulation of inhibitors of canonical Wnt signaling [13], it is plausible to assume that the mechanical stress-induced increase in IL-11 expression constitutes an important signaling cascade mediating the mechanical stress signal to the enhancement of osteoblast differentiation.

The present study demonstrated that phosphorylated BR-Smads bind to the C-terminal region of JunD as well as to SBE of the *IL-11* gene promoter. Our previous results demonstrated that ΔFosB/JunD heterodimer binds to the AP-1 site on the *IL-11* gene promoter [13]. These results suggest that phosphorylated BR-Smads form complex with ΔFosB/JunD heterodimer on the IL-11 promoter with binding of BR-Smads to the SBE and of ΔFosB/JunD heterodimer to the AP-1 site, and that they form a complex with other transcription co-factors to enhance *IL-11* gene transcription. The mechanism whereby the formation of such transcription factor complex enhances IL-11 gene transcription remains to be clarified.

In conclusion, the present observations demonstrate that PKCδ-Smad1 pathway plays an important role in the intracellular signaling in response to mechanical stress, and that a cross-talk between PKCδ-Smad1 and ERK-CREB-ΔFosB/JunD pathways synergistically stimulates osteogenic program in response to mechanical stress in osteoblasts.

## Materials and Methods

### Ethics Statement

All experiments with animals were performed according to the guidelines for animal protection in the University of Tokushima, and approved by the Animal Experiments Committee, Tokushima University (#Toku-Animal06121) for animal protection.

### Materials

Recombinant human transforming growth factor -β1 and recombinant human BMP-2 were purchased from Peprotech Inc. (Rocky Hill, NJ). Recombinant human PKCδ was purchased from Calbiochem (San Diego, CA). Calphostin C (PKC inhibitor), U0126 (mitogen-activated protein kinase kinase inhibitor), SB203580 (p38 kinase inhibitor), LY294002 (PI3K inhibitor), H89 (PKA inhibitor), Chelerythrine chloride (PKC inhibitor), Go6976 (PKC inhibitor) and Bisindolylmaleimide I (PKC inhibitor) were purchased from Calbiochem. TPA (PKC activator) was purchased from Sigma (St Louis, MO).

Antibodies against phospho-Smad1 which recognizes Ser463/465-phosphorylated Smad1/5/8, against phospho-Smad2 which recognizes Ser465/467-phosphorylated Smad2/3, and Smad2 were purchased from Cell Signaling Technology (Berverly, MA). Antibody against Smad1 was purchased from MILLIPORE Corp. (Bedford, MA). Antibody against β-actin was purchased from Sigma. Antibody against phospho-Serine/Threonine (pSer/Thr) was purchased from Invitrogen (Carlsbad, CA). Antibody against phospho-Tyrosine (pTyr), PKCδ, PKCε, PKCη and PKCθ were purchased from Santacruz Biotechnology (San Diego, CA). Antibody against Phospho-PKCδ..Tyr311 ˙ was purchased from Stressgene (Victoria, Canada). Anti-Flag antibody was purchased from Sigma. Anti-V5 antibody was purchased from Invitrogen. Anti-Myc antibody was purchased from Santacruz Biotechnology.

All chemicals for dual-luciferase assays were purchased from Promega (Madison, WI). The other chemical reagents were purchased from Sigma unless otherwise specified.

### Cell culture and *in vitro* mechanical loading by FSS

Primary osteoblasts were prepared from calvaria of newborn mice by sequential digestion as previously described [14]. Isolated mPOBs were cultured in α minimum essential medium (αMEM; Invitrogen) supplemented with 10% fetal bovine serum (FBS; MBL Co., Ltd, Nagoya, Japan), penicillin/streptomycin (Invitrogen) for 48 hours. Before experiments, 1×10^5^ cells/ml mPOBs were seeded on culture dishes, grown to 70–80% confluence, and serum-deprived inαMEM with 1% FBS for 24 hours. As reported previously, these mPOBs can undergo osteoblastic differentiation *in vitro* to become osteogenic cells [14].

For mechanical loading *in vitro*, cells were exposed to FSS by placing 6-well culture dishes (35 mm diameter) on a horizontal rotating platform with rotating radius of 9 mm fixed inside the culture incubator and rotated at 120 rpm [14]. This horizontally rotating apparatus creates rotating fluid flow of culture medium in culture dishes. In a cone viscometer, because the angle of cone is constant, the FSS to cells is almost constant in most part of culture dishes [31], whereas the fluid surface height is not proportional to the distance from the center in our system. In our apparatus, the cycles of rotator and the fluid were almost synchronized, and the fluid surface height at the edge is the highest where rotation of them is in the same direction while the surface height at the edge is the lowest where rotation of them is in the opposite direction. Therefore, the height of the fluid (*z*) ranged as follows:
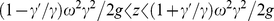
Where *ω* is the angular velocity, *r* is the radius of culture dish, *r′* is the radius of rotator, and *g* is the gravity forces (9.8 m/s^2^). Because *r′* is almost 1/2 of *r*, *z* is approximately 1.4 mm at the lowest edge and 4.0 mm at the highest edge. The *z* values in our system correspond to *r* tan*α* in a cone viscometer. Therefore, hypothetical fluid surface angles (*α*) on the line of the lowest ridge can be calculated by the following formula:
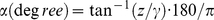
where *z* is the minimal value on the lowest ridge, and *r* is the distance from the center.

Based upon the calculated FSS (*τ*) of 2.0 Pa from the Reynolds number (*R*) and the following formulas in Reference 31:
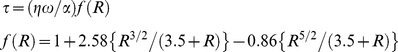

*τ* can be calculated as follows:
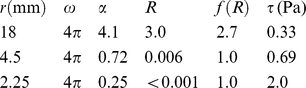
Thus, FSS in 6-well culture dish is expected to range from 0.33 to 2.0 Pa.

The above model may not be able to precisely estimate FSS at the very edge of the dish where the fluid contacts with the outer wall, and at the very center of the well. However, based on the calculations above, we expect that FSS in our system mostly remains within the physiological range of 2.0 Pa or lower [32].

### Adenovirus Infection

All recombinant adenovirus used in this study (Flag-Smad1, Flag-Smad5, and Flag-Smad8) were reported previously [22], and high-titered stocks of recombinant adenoviruses were grown in HEK293 cells (Riken Bioresource Center, Ibaragi, Japan). For experiments, mPOBs passage two were infected with various recombinant adenoviruses at a multiplicity of infection (m.o.i) varying from 0 to 100. At 48 h after infection, cells were harvested and examined for expression or phosphorylation of Smads by Western blot analysis.

### GST pull-down assay

GST-Smad1 plasmids were transformed into BL21 competent cells (Invitrogen) and the protein expression was induced by the addition of isopropyl-1-thio-b-D- galactopyranoside (IPTG) and then bacterial pellets were lysed by freeze-thaw cycles and sonication. GST fusion protein then were mixed with Glutathione-Sepharose (GE Healthcare Life Sciences, Little Chalfont, Buckinghamshire, UK) and incubated at 4C° for 1 hour. The Sepharose beads were then washed three times and used for GST pull-down assays. mPOBs were lysed in lysis buffer (20 mM HEPES-KOH, pH7.5, 150 mM NaCl, 1% Triton-X100, 0.5 mM phenylmethylsulfonyl fluoride (PMSF), and 1× protease inhibitor cocktail (SIGMA)) and aliquots of the lysate were then incubated under constant agitation for 1 hour at 4C° with GST fusion protein coupled to Glutathione-Sepharose. Subsequently, complexes were washed three times and then bound proteins were eluted and separated on SDS-PAGE and detected by Western Blots using appropriate antibodies.

### Protein analysis

Preparation of nuclear extracts or total cell lysates were prepared as described before [14]. For Western blot analysis, 30 µg of protein was separated on a SDS-PAGE (10–20% gradient gel, ATTO, Tokyo, Japan) and transferred to PVDF membrane (MILLIPORE). The membrane was rinsed and blocked with 5% non-fat skim milk in Tris-buffered saline (TBS) with 0.1% Tween-20 for 1 hour at room temperature, then blotted sequentially with primary antibodies, then with a horseradish peroxidase-conjugated secondary antibody (Promega) for 1 hour, and the protein bands were visualized with an ECL-Plus detection system (GE Healthcare Life Sciences).

### Plasmid construction

Myc-tagged Smad1 used in this study was reported previously [22]. A cDNA encoded from mouse V5-tagged ΔFosB (full-length), Flag-tagged JunD (WT: full-length, Δ1: 1–344a.a., Δ2: 1–321a.a.), and V5-tagged PKCδ (WT: full-length) were amplified by polymerase chain reaction (PCR) using PrimeSTAR (TaKaRa, Shiga, Japan) and subcloned into the mammalian expression vector using pcDNA3.1D Directional TOPO Expression Kit (Invitrogen) as referred to the manufacturer's instructions. A PKCδ mutant (PKCδ Y311F) was generated by site-directed mutagenesis using the QuickChange™ Site-Directed Mutagenesis Kit (STRATAGENE, La Jolla, CA) with mutagenic primers (sense: 5′- CAGAGTCTGTCGGAATATTCCAGGGATTTGAGAAGAA -3′, antisense: 5′- TTCTTCTCAAATCCCTGGAATATTCCGACAGACTCTG-3′). These expression plasmids were checked and verified sequencing (ABI model 377, Applied Biosystems) promoted by Support Center for Advanced Medical Societies, University of Tokushima.

### Mouse *IL-11* promoter construction

Fragments of mouse *IL-11* gene (GeneBankTM number AY225468) upregulatory region from nucleotides −269 to −6 (−280wt) [33] were cloned and subcloned into a luciferase reporter plasmid pGL3-basic (Promega), which lacks eukaryotic promoter and enhancer sequences (with the transcription start site being numbered +1). A series of mutant plasmids (−2805'Am, −280Sm, and −2805'Am/Sm) were generated by site-directed mutagenesis with mutagenic primers including 5′- ACTCtca -3′ (WT: 5′- tgactca -3′, position at −251 to −245) for 5′AP-1 mutation and 5′- TTTgcc -3′ (WT: 5′- cccgccc -3′, position at −201 to −196) for SBE mutation, respectively. Capital letters indicate substituted nucleotides that disrupt AP-1 and Smad binding sites.

### Gene silencing

Duplexed siRNA was designed and synthesized by Hokkaido System Sciences (Sapporo, Japan). The siRNA sequences for targeting mouse fosB/δfosB and mouse PKCδ are as follows; mouse fosB/δfosB: 5′- UCUCUUUACACACAGUGAAGUUCAA -3′ and mouse PKCδ: 5′-AGCUGAAGGGCAAAGAUAAGUACUUAG-3′. Scrambled siRNA was synthesized and used as a negative control. Transfection of the siRNA oligonucleotides was performed using Lipofectamine RNAiMAX (Invitrogen) in opti-MEM (Invitrogen) without serum. Gene silencing efficiency was evaluated by Western blotting using specific antibody for targeting gene products.

### Measurement of transcriptional activity

mPOBs were seeded in 6-well culture plates at 50% confluence and co-transfected with 2 µg of chimeric Firefly luciferase reporter plasmid combined with Renilla luciferase reporter plasmid (pRL-TK, Promega) as a control, and/or with mammalian expression plasmids using GenePORTER 2 Transfection Reagent (Genlantis, San Diego, CA) in opti-MEM (Invitrogen) supplemented with 1% serum. Sixteen hours after transfection, the medium was replaced by αMEM containing with 1% FBS and treated with various reagents and exposed to FSS by placing the culture plates on a shaking apparatus at 100 to 120 rpm. For dual luciferase assay, the cells were washed and lysed with passive cell lysis buffer (Promega) and both Firefly and Renilla luciferase activity was measured with luminometer (ATTO) by mixing 50 µl of luciferase substrate solution (Promega) with 10 µl of cleared cell lysates. Transcriptional activity was corrected for Renilla luciferase activity or protein concentrations.

### Measurement of protein kinase activity

Cells were stimulated with different agents and then lysed with a lysis buffer for the kinase assay (20 mM Tris-HCl, pH 7.5, 137mM NaCl, 1 mM EDTA, 1 mM EGTA, 10% glycerol, 1% NP-40, 1 mM sodium orthovanadate (Na_3_VO_4_), 4.5 mM sodium pyrophosphate, 47.6 mM sodium fluoride (NaF), 9.26 mM β-glycerophosphate, 0.5 mM dithiothreitol (DTT) and 1× protease inhibitor cocktail). Lysates were then immunoprecipitated using appropriate antibodies. Kinase assays were performed in the immunoprecipitates using the PKC assay kit (Upstate Biotechnology, Lake Placid, NY) following the directions provided by the company with minor modifications. Briefly, Immunoprecipitaes bound to agarose beads were resuspended in assay dilution buffer containing phospatidylserine and diacylglycerol. The kinase reaction was initiated either the addition of a Mg^2+^/adenosine triphosphate (ATP) mixtures (75 mM MgCl_2_ and 0.5 mM ATP) containing 10 mCi of [γ-^32^P]ATP (3,000 Ci/mmol) and the substrate (GST-Smad1 or histone H1 as a control). The reaction mixtures were briefly vortexed and then incubated at 30C° for 30 min with occasional mixing. After incubation, the kinase reaction was terminated by adding 4× sample buffer and boiling the samples. Samples were resolved using 4–20% SDS-PAGE gels and then dried and analyzed by autoradiography.

### DNA precipitation assay

Biotinylated double-stranded oligonucleotide probes containing tandem AP-1 of the mouse IL-11 promoter sequences [33] were prepared by annealing complementary oligonucleotides followed by ethanol precipitation. Then annealed DNA probes were incubated with equal amounts of nuclear extracts and poly dI-dC (GE Healthcare LifeSciences) in reaction buffer (20 mM HEPES-KOH pH 7.4, 0.1 M NaCl, 0.5 mM EDTA, 0.5 mM EGTA, 0.5 mM DTT, 1 mM PMSF, 1× protease inhibitor cocktail, 20 mM NaF, and 1 mM Na_3_VO_4_) for 4C° with gentle agitation, and then mixed with Streptavidine Sepharose beads (GE HealthCare LifeSciences) at 4C° for 30 min. DNA-protein complexes bound to beads were collected by centrifugation and washed with ice-cold reaction buffer for 4 times. After the final wash, beads were collected and resuspended in 2× sample buffer and boiled. DNA-bound proteins were separated on a SDS-PAGE and detected by Western blot analysis.

### Statistical analysis

Values for parameters within a group are expressed as means ± S.E.M. For comparisons among groups, statistical significance was assessed using an unpaired two-tailed Student's *t* test for two-group comparisons or one-way ANOVA, and the significance of each difference was determined by post-hoc testing using the Tukey-Kramer method. These analyses were performed on an Apple Macintosh computer with the use of Excel (Microsoft) and StatView statistical package (StatView 5.0; SAS Institute Inc.). A *P* value of less than 0.05 was considered significant.

## Supporting Information

Figure S1 IL-11 expression is enhanced by BMP-2. mPOBs were treated with 300 ng/ml BMP-2 for the indicated period of time, and quantitative real-time PCR analysis was performed using the SYBR Green chemistry. The reaction mixture (20 µl) contained 200 nM PCR primers sets for IL-11 (forward: 5′- aaattcccagctgacggagatcac -3′, reverse: 5′- tacatgccggaggtaggacatcaa -3′ ) and GAPDH (forward: 5′- ggcaaattcaacggcacagtca -3′, reverse: 5′- ggcaaattcaacggcacagtca -3′), 10 µl SYBR premix Ex Taq (TaKaRa, Shiga, Japan) and cDNA templates equivalent to 100 ng total RNA.
(8.70 MB TIF)

Figure S2 Mechanical stress enhances IL-11 but not BMP-2 expression. mPOBs were exposed to FSS for the indicated period of time, and then quantitative real-time PCR analysis was performed using the SYBR Green chemistry. The reaction mixture (20 µl) contained 200 nM PCR primers sets for IL-11 (forward: 5′- aaattcccagctgacggagatcac -3′, reverse: 5′- tacatgccggaggtaggacatcaa -3′ ), BMP-2 (forward: 5′- cgcagcttccatcacgaaga -3′, reverse: 5′- tgttcccggaagatctggagtt -3′), and GAPDH (forward: 5′- ggcaaattcaacggcacagtca -3′, reverse: 5′- ggcaaattcaacggcacagtca -3′), 10 µl SYBR premix Ex Taq (TaKaRa, Shiga, Japan) and cDNA templates equivalent to 100 ng total RNA. Data are means ± S.E.M. for four experiments, and difference from non-treated cells was analyzed by one-way ANOVA. *P<0.05 compared with non-treated cells.
(8.70 MB TIF)
