# Supplementary figures and images for: Mechanical Stress Activates Smad Pathway through PKCδ to Enhance Interleukin-11 Gene Transcription in Osteoblasts
Source: PLoS One. 2010 Sep 29;5(9):e13090. doi: 10.1371/journal.pone.0013090 (PMC2947522; doi:10.1371/journal.pone.0013090)

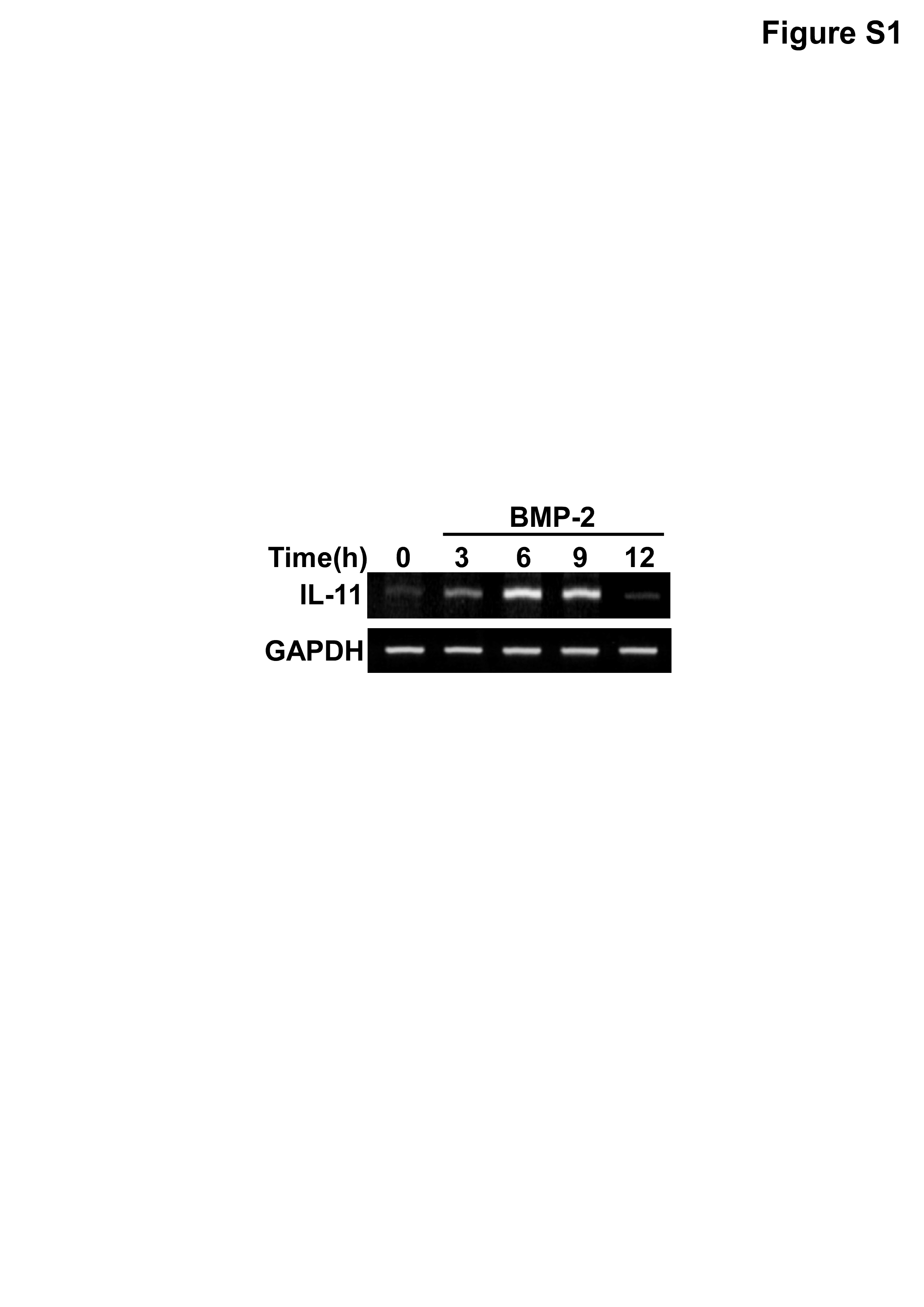

Supplement: Figure S1 — IL-11 expression is enhanced by BMP-2. mPOBs were treated with 300 ng/ml BMP-2 for the indicated period of time, and quantitative real-time PCR analysis was performed using the SYBR Green chemistry. The reaction mixture (20 µl) contained 200 nM PCR primers sets for IL-11 (forward: 5′- aaattcccagctgacggagatcac -3′, reverse: 5′- tacatgccggaggtaggacatcaa -3′ ) and GAPDH (forward: 5′- ggcaaattcaacggcacagtca -3′, reverse: 5′- ggcaaattcaacggcacagtca -3′), 10 µl SYBR premix Ex Taq (TaKaRa, Shiga, Japan) and cDNA templates equivalent to 100 ng total RNA. (8.70 MB TIF) [file pone.0013090.s001.tif]

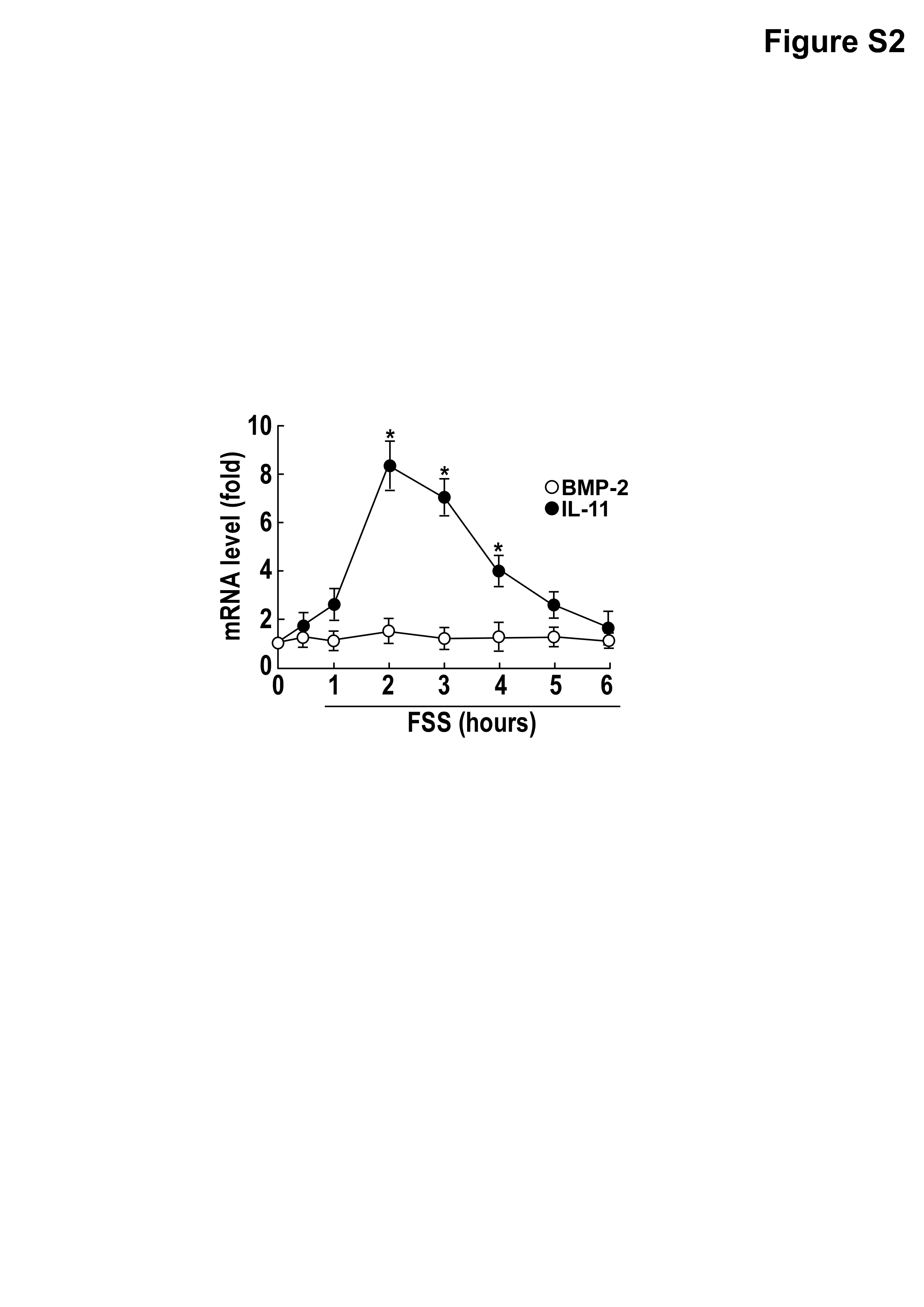

Supplement: Figure S2 — Mechanical stress enhances IL-11 but not BMP-2 expression. mPOBs were exposed to FSS for the indicated period of time, and then quantitative real-time PCR analysis was performed using the SYBR Green chemistry. The reaction mixture (20 µl) contained 200 nM PCR primers sets for IL-11 (forward: 5′- aaattcccagctgacggagatcac -3′, reverse: 5′- tacatgccggaggtaggacatcaa -3′ ), BMP-2 (forward: 5′- cgcagcttccatcacgaaga -3′, reverse: 5′- tgttcccggaagatctggagtt -3′), and GAPDH (forward: 5′- ggcaaattcaacggcacagtca -3′, reverse: 5′- ggcaaattcaacggcacagtca -3′), 10 µl SYBR premix Ex Taq (TaKaRa, Shiga, Japan) and cDNA templates equivalent to 100 ng total RNA. Data are means ± S.E.M. for four experiments, and difference from non-treated cells was analyzed by one-way ANOVA. *P<0.05 compared with non-treated cells. (8.70 MB TIF) [file pone.0013090.s002.tif]
